# In Memory of Hagop Akiskal

**DOI:** 10.2174/1745017902117010048

**Published:** 2021-05-31

**Authors:** Mauro G. Carta, Francesc Colom, Andreas Erfurth, Michele Fornaro, Heinz Grunze, Elie Hantouche, Antonio E. Nardi, Antonio Preti, Eduard Vieta, Elie Karam

**Affiliations:** 1 Department of Medical Sciences and Public Health, University of Cagliari, Cagliari, Italy; 2 Hospital del Mar, Barcelona, Spain; 3 Department of Psychiatry and Psychotherapeutic Medicine, Klinik Hietzing, Vienna, Austria; 4 Laboratory of Molecular and Translational Psychiatry, Department of Neuroscience, School of Medicine, University "Federico II", Naples, Italy; 5 Center for Psychiatry Weinsberg, Klinikum am Weissenhof, Weinsberg, Germany; 6 Center for Anxiety and Mood Disorders, Anxiety & Mood Center, 117, Rue de Rennes, Paris 75006, France; 7 Institute of Psychiatry,Federal University of Rio de Janeiro,Rio de Janeiro, Brazil; 8 Department of Neurosciences, University of Turin, 10124 Turin, Italy; 9 Bipolar Disorders Unit, Hospital Clinic, Institute of Neurosciences, University of Barcelona, IDIBAPS, CIBERSAM, Barcelona, Catalonia, Spain; 10 Department of Psychiatry and Clinical Psychology, Faculty of Medicine, Balamand University, Beirut, Lebanon

The death of Hagop Akiskal left a great void in all who knew him, and many of us knew him, indeed, all over the world.

Hagop was an attentive clinician and innovative researcher as well as a mentor who accompanied the growth of many young psychiatrists, a researcher sometimes controversial, never predictable, whose reflections have changed the way of seeing and conceiving mental illness in recent decades. And, above all, a gifted visionary that changed the field of bipolar disorders for better and forever. He rawarded of several international prizes, including the Jean Delay Prize of the World Psychiatric Association in 2002 (perhaps the most prestigious award in the field of psychiatry).

In our cultures when a great friend dies the village elderly gather, talk about him and celebrate him or her. Without even hiding his or her weaknesses. We are old, in our own cultures of the global village of psychiatry, and we are his/her friends. We are chatting with you, friends of the global village of psychiatry and mental health, chatting about the life of Hagop Akiskal.

1) First the cultural roots of Hagop.

Globalization in the world of science has led to a system that can be accessed from any terminal. If you live in an unheard unheard-of country, you could write an editorial for NEJM. However, the creation of dominant currents of thought often leads to disregarding the sometimes-essential contribution of cultural spheres unknown to those who are leaders at that time. For the researcher, young or old, coming from the periphery of the world, it is not always easy to deal with the top scientific centers. Akiskal was an exception; he never, never declined to reply to an email sent by a young researcher from anywhere in the world. Hagop always encouraged the ideas he thought were interesting. And he always remembered those contacts. He kept in touch with them to oversee their research and how their ideas were evolving. If you look at the list of his publications, you will be struck with the variety of researchers he worked with: from the Balkans to Brazil, from China and Japan to France, from Australia to Lebanon, and of course with researchers from the USA, and Italy. In a famous interview with Paula Clayton in 2008, Akiskal says that he learned a lot from the Italians, but when you look closely at the names of the Italians with whom he published most frequently on PubMed, it turns out that these are two mostly two (at that time) *young* psychiatrists (Perugi and Benazzi). His generosity in responding to young people, to the point of being even criticized for being too generous as an Editor of an important Journal, perhaps had a deep root: he said once that he rarely saw anyone smiling in his family because of the ordeals they had lived; his reaction was to offer hope.

The cultural roots of Hagop Akiskal, an Armenian whose family survived the Genocide and adopted Lebanon as the new country, matured in that melting pot of cultures that Beirut was during the fifties and sixties of the last century. Hagop was drawn to journalism from an early age; But that's his big- mother who firmly dissuaded him by specifying that this job could expose an Armenian refugee to great risk and, therefore, urged him to choose a more secure profession, like medicine. This inner fiber has always echoed in Hagop's writings and lectures. A true gift of “Sharing” his knowledge.

On the other hand, its origin and roots are fundamental to understand his contribution. Hagop spoke six languages, including Turkish, Arabic, and French. He knew the history of Latin, Greek, and Arab medicine; he was familiar with Kraepelin and 19th 19th-century French psychopathology which was introduced by his early Lebanese mentors. It is largely due to his contribution that sources of knowledge were revived in America, at a time in which the classical European tradition of psychopathology was largely unknown.

2) The second point is the concepts of Spectrum and Temperament. Akiskal introduced two concepts, at times thought by some of not being well defined, but yet and unmistakably proved to be quite fertile: The first is the spectrum [1, 2]. He was convinced that (almost) all mood disorders have the same vulnerability matrix and, in agreement with Attanasio Kukopulos, that the primacy in psychopathology is mania [3, 4]. This concept, an echo to Kraepelin, should have been denied by the DSM-5, a manual that clearly splits depression from bipolar disorder. Dichotomy which still represents the source of vivid debate.

The second concept is that of temperament [1, 5]. The spectrum has “-under threshold” areas. Having sub-threshold characteristics and/or temperament, without full-blown disorders, could sometimes be maladaptive, or present elements of paradoxical adaptivity. Mixing these two aspects could simplistically lead to a vision of the psychiatrist as in “Dr. Knock and the triumph of medicine” [6]: if you pathologize the temperament heavily (and many are temperamentally “bipolar”) you will find some idiot who will think it is appropriate to put lithium in the water pipes. However, if we reflect very well on the profound implications of these concepts, a fundamentally evolutionary vision of the bipolar spectrum emerges that can predict how the alleged basic vulnerability can also represent a potential advantage in some situations as in megacities “in which life is expected to run 24 hours a day for seven days a week” [7], a point one of us had discussed thoroughly with Hagop. The presence of “dilute” bipolar genes in the population and the reason why such genes remain in the population, is deeply related to the evolutionary nature of human beings, as exemplified by themes such as creativity and leadership, extraversion and openness, which may span across adaptive and functional advantages, to impairing gradients leading to overt mental illness and social disadvantages.

From an epidemiological point of view, the hypothesis of an adaptive side of hyperactivity may explain how the prevalence of mood disorders in Western society might be indeed elevated [8, 9] although cross-national differences in prevalence of mental disorders are far from being settled as emerged from the World Mental Health Survey Initiative on which some of us are working for a long time [10]. Nevertheless, the interpretation of bipolar disorders in the evolutionary key, it might be useful to counter the stigmatizing vision of mood disorders and bipolar disorders in particular.

It was an important step by Hagop not only to recognize temperament as the end point of Kraepelin's continuum from “basic state” to “manic-depressive insanity” as a historically significant concept, but also to translate this hypothesis into the present day and to test it scientifically. The TEMPS scale he and his coworkers developed to assess temperament has been translated and validated in a vast number of languages [11-18], including studies on the construction of a brief version [19-24]. The use of the TEMPS seems particularly important in the definition of comorbidities in the bipolar spectrum [25-28].

3) Finally, the third point concerns Akiskal's undisputed moral integrity never touched of the controversies that have characterized the relationship between, scientific research and economic interests. Some concepts introduced by Hagop, as often happens in innovation, have opened up a new style of care and therefore benefits for people but undoubtedly opened up market areas for the pharma industry. For example, the dropping of the concept of neurotic depression and the introduction of the diagnosis of Dysthymia. The revolution that led to a “unitary” vision of depression came through a long journey in Akiskal's thought. In fact, a work published in Science in 1973 by a very young Hagop with William McKinney, entitled “Depressive disorders: toward a unified hypothesis” [29], played a decisive role in leading to the “unitary” approach to depression of the DSM-III, notably different from that of the Research Diagnostic Criteria. However, to read that work carefully, his perspective was far from a simplistically “biological” view as opposed to an equally simplistically “psychogenetic” view of depression. His was a very coherent perspective to that complex vision of the mind that was imposing in those years through a new synthesis that made use of the contributions of the ethological reading of psychopathology [30], of the new cognitive-behavioral psychology [31] and neurophysiology [32].

However, the loss of importance of neurotic depression was signed by the progressive waning of the relevance of the context in which mood symptoms occur. This approach was even reinforced in DSM-5, wich, based on field studies [33] no longer considers bereavement as a criterion for excluding the diagnosis of depressive episodes [DSM-5 APA 2013]. The new perspective had relevant consequences on clinical practice: an observational study on Liaison Psychiatric in US general hospitals found from 1988 to 1997 the percentage of diagnosis of Major Depression with concomitant medical illness increased from 6.4% to 14.7% while the diagnosis of Adjustment Disorder with Depressed Mood decreased from 28% to 14.7% [34]. It is not difficult to observe that in the same years there was a surge in the market for antidepressants [35, 36].

In the years immediately following the rise of the concept of the bipolar spectrum, but not necessarily only due to it, the market for mood stabilizers opens up, as well as the newly synthesized antipsychotics which increasingly were assessed and promoted for their mood-stabilizing properties. Although Hagop was one of the epigones of this revolution, however, his figure, so outside the schemes of power, even so difficult to exploit, cannot be said to have been “used” by those who have exploited the economic benefits of the new market. Indeed, his convictions and his frankness have often not made him a friend of the powerful world of the pharmaceutical industry. This awareness and pride of the importance of his role as a healer and researcher “above everything and everyone”; even when confronted with easy benefits made Hagop’s legacy and memory even fonder.

Hagop was an incredible educator and speaker. Whenever he was giving a speech at a congress, the room was full way ahead of time. We all remembered rooms and corridors overcrowded with colleagues that would not miss a new Hagop talk…even if they listened to the “same talk” a month before: Hagop was always creative and always inspired, as any brilliant free-jazz soloist would, never repeated the same piece. There was always something to be learned or to discuss passionately. He loved the interaction with the public, especially with clinicians. Particularly passionate with the younger ones. His deep clinical talent was obvious when he was presenting data. He did not like the numbers as much as he liked the patients and the clinical knowledge to be spread through his inspiring talks, which had a profound impact on many of us. Large international studies have validated many of his theories [37] and his approach is still under debate as regards the classification of mental disorders [38, 39] and their management [40].

We are so happy to have had the chance to work with Hagop, and to have witnessed his unique combination of knowledge, reflection and clinical acumen being coupled with courage and ethics over the years. Hagop you are deeply missed and as you can tell, our connections and conversations will continue beyond the limits of time.

## References

[1] Akiskal H.S., Cassano G.B., Musetti L., Perugi G., Tundo A., Mignani V. (1989). Psychopathology, temperament, and past course in primary major depressions. 1. Review of evidence for a bipolar spectrum.. Psychopathology.

[2] Akiskal H.S., Weise R.E. (1992). The clinical spectrum of so-called “minor” depressions.. Am. J. Psychother..

[3] Benazzi F., Koukopoulos A., Akiskal H.S. (2004). Toward a validation of a new definition of agitated depression as a bipolar mixed state (mixed depression).. Eur. Psychiatry.

[4] Koukopoulos A., Ghaemi S.N. (2009). The primacy of mania: A reconsideration of mood disorders.. Eur. Psychiatry.

[5] Akiskal H.S., Hirschfeld R.M., Yerevanian B.I. (1983). The relationship of personality to affective disorders.. Arch. Gen. Psychiatry.

[6] Romains J. Knock, ou, Le triomphe de la médecine 2004, Gallimard (reprint fist edition 1925).

[7] Carta M.G., Preti A., Akiskal H.S. (2018). Coping with the new Era: Noise and light pollution, hperactivity and steroid hormones. towards an evolutionary view of bipolar disorders.. Clin. Pract. Epidemiol. Ment. Health.

[8] Compton W.M., Conway K.P., Stinson F.S., Grant B.F. (2006). Changes in the prevalence of major depression and comorbid substance use disorders in the United States between 1991-1992 and 2001-2002.. Am. J. Psychiatry.

[9] Goldney R.D., Eckert K.A., Hawthorne G., Taylor A.W. (2010). Changes in the prevalence of major depression in an Australian community sample between 1998 and 2008.. Aust. N. Z. J. Psychiatry.

[10] Demyttenaere K., Bruffaerts R., Posada-Villa J., Gasquet I., Kovess V., Lepine J.P., Angermeyer M.C., Bernert S., de Girolamo G., Morosini P., Polidori G., Kikkawa T., Kawakami N., Ono Y., Takeshima T., Uda H., Karam E.G., Fayyad J.A., Karam A.N., Mneimneh Z.N., Medina-Mora M.E., Borges G., Lara C., de Graaf R., Ormel J., Gureje O., Shen Y., Huang Y., Zhang M., Alonso J., Haro J.M., Vilagut G., Bromet E.J., Gluzman S., Webb C., Kessler R.C., Merikangas K.R., Anthony J.C., Von Korff M.R., Wang P.S., Brugha T.S., Aguilar-Gaxiola S., Lee S., Heeringa S., Pennell B.E., Zaslavsky A.M., Ustun T.B., Chatterji S., WHO World Mental Health Survey Consortium (2004). Prevalence, severity, and unmet need for treatment of mental disorders in the World Health Organization World Mental Health Surveys.. JAMA.

[11] Fountoulakis K.N., Siamouli M., Magiria M., Pantoula E., Moutou K., Kemeridou M., Mavridou E., Panagiotidis P., Loli E., Batsiari E., Preti A., Tondo L., Gonda X., Rihmer Z., Akiskal K., Akiskal H. (2014). Standardization of the TEMPS-A in the Greek general population.. J. Affect. Disord..

[12] Figueira M.L., Caeiro L., Ferro A., Severino L., Duarte P.M., Abreu M., Akiskal H.S., Akiskal K.K. (2008). Validation of the Temperament Evaluation of Memphis, Pisa, Paris and San Diego (TEMPS-A): Portuguese-Lisbon version.. J. Affect. Disord..

[13] Pompili M., Girardi P., Tatarelli R., Iliceto P., De Pisa E., Tondo L., Akiskal K.K., Akiskal H.S. (2008). TEMPS-A (Rome): psychometric validation of affective temperaments in clinically well subjects in mid- and south Italy.. J. Affect. Disord..

[14] Vázquez G.H., Nasetta S., Mercado B., Romero E., Tifner S., Ramón Mdel.L., Garelli V., Bonifacio A., Akiskal K.K., Akiskal H.S. (2007). Validation of the TEMPS-A Buenos Aires: Spanish psychometric validation of affective temperaments in a population study of Argentina.. J. Affect. Disord..

[15] Karam E.G., Mneimneh Z., Salamoun M., Akiskal K.K., Akiskal H.S. (2005). Psychometric properties of the Lebanese-Arabic TEMPS-A: A national epidemiologic study.. J. Affect. Disord..

[16] Karam E.G., Mneimneh Z.N., Salamoun M.M., Akiskal H.S., Akiskal K.K. (2007). Suitability of the TEMPS-A for population-based studies: Ease of administration and stability of affective temperaments in its Lebanese version.. J. Affect. Disord..

[17] Sánchez-Moreno J., Barrantes-Vidal N., Vieta E., Martínez-Arán A., Saiz-Ruiz J., Montes J.M., Akiskal K., Akiskal H.S. (2005). Process of adaptation to Spanish of the Temperament Evaluation of Memphis, Pisa, Paris and San Diego Scale. Self applied version (TEMPS-A).. Actas Esp. Psiquiatr..

[18] Akiskal H.S., Akiskal K., Allilaire J.F., Azorin J.M., Bourgeois M.L., Sechter D., Fraud J.P., Chatenêt-Duchêne L., Lancrenon S., Perugi G., Hantouche E.G. (2005). Validating affective temperaments in their subaffective and socially positive attributes: Psychometric, clinical and familial data from a French national study.. J. Affect. Disord..

[19] Erfurth A., Gerlach A.L., Hellweg I., Boenigk I., Michael N., Akiskal H.S. (2005). Studies on a German (Münster) version of the temperament auto-questionnaire TEMPS-A: Construction and validation of the briefTEMPS-M.. J. Affect. Disord..

[20] Erfurth A., Gerlach A.L., Michael N., Boenigk I., Hellweg I., Signoretta S., Akiskal K., Akiskal H.S. (2005). Distribution and gender effects of the subscales of a German version of the temperament autoquestionnaire briefTEMPS-M in a university student population.. J. Affect. Disord..

[21] Preti A., Corrias I., Gabbrielli M., Lai V., Muratore T., Pintus E., Pintus M., Sanna S., Scanu R., Tronci D., Vellante M., Siddi S., Petretto D.R., Carta M.G. (2015). The independence of schizotypy from affective temperaments--a combined confirmatory factor analysis of SPQ and the short TEMPS-A.. Psychiatry Res..

[22] Preti A., Vellante M., Gabbrielli M., Lai V., Muratore T., Pintus E., Pintus M., Sanna S., Scanu R., Tronci D., Corrias I., Petretto D.R., Carta M.G. (2013). Confirmatory factor analysis and measurement invariance by gender, age and levels of psychological distress of the short TEMPS-A.. J. Affect. Disord..

[23] Preti A., Vellante M., Zucca G., Tondo L., Akiskal K., Akiskal H. (2010). The Italian version of the validated short TEMPS-A: The temperament evaluation of Memphis, Pisa, Paris and San Diego.. J. Affect. Disord..

[24] Woodruff E., Genaro L.T., Landeira-Fernandez J., Cheniaux E., Laks J., Jean-Louis G., Nardi A.E., Versiani M.C., Akiskal H.S., Mendlowicz M.V. (2011). Validation of the Brazilian brief version of the temperament auto-questionnaire TEMPS-A: The brief TEMPS-Rio de Janeiro.. J. Affect. Disord..

[25] Vyssoki B., Blüml V., Gleiss A., Friedrich F., Kogoj D., Walter H., Zeiler J., Höfer P., Lesch O.M., Erfurth A. (2011). The impact of temperament in the course of alcohol dependence.. J. Affect. Disord..

[26] Salem B.A., Vyssoki B., Lesch O.M., Erfurth A. (2014). Lesch typology and temperament in opioid dependence: a cross-sectional study.. J. Affect. Disord..

[27] Schiele M.A., Herzog K., Kollert L., Böhnlein J., Repple J., Rosenkranz K., Leehr E.J., Ziegler C., Lueken U., Dannlowski U., Pauli P., Arolt V., Zwanzger P., Deckert J., Erfurth A., Domschke K. (2020). Affective temperaments (TEMPS-A) in panic disorder and healthy probands: Genetic modulation by *5-HTT* variation.. World J. Biol. Psychiatry.

[28] Skala K., Kapusta N.D., Schlaff G., Unseld M., Erfurth A., Lesch O.M., Walter H., Akiskal K.K., Akiskal H.S. (2012). Suicidal ideation and temperament: An investigation among college students.. J. Affect. Disord..

[29] Akiskal H.S., McKinney W.T. (1973). Depressive disorders: Toward a unified hypothesis.. Science.

[30] McKinney W.T., Hanin I., Usdin E. (1977). Biobehaviorial models of depression in monkeys.. Animal models in psychiatry..

[31] Beck A. (1967). Depression: Causes andTreatment..

[32] Eclles JC (1973). The understanding of the Brain..

[33] Karam E.G., Tabet C.C., Alam D., Shamseddeen W., Chatila Y., Mneimneh Z., Salamoun M.M., Hamalian M. (2009). Bereavement related and non-bereavement related depressions: A comparative field study.. J. Affect. Disord..

[34] Diefenbacher A, Strain JJ (2002). Consultation-liaison psychiatry: Stability and change over a 10-year period.. Gen Hosp Psychiatry.

[35] Carta M.G., Kovess V., Hardoy M.C., Brugha T., Fryers T., Lehtinen V., Xavier M. (2004). Psychosocial wellbeing and psychiatric care in the European Communities: Analysis of macro indicators.. Soc. Psychiatry Psychiatr. Epidemiol..

[36] Carta M.G., Angermeyer M.C. (2015). The triumph of the DSM and patient-centered psychiatry.. Cult. Med. Psychiatry.

[37] Angst J., Gamma A., Neuenschwander M., Ajdacic-Gross V., Eich D., Rössler W., Merikangas K.R. (2005). Prevalence of mental disorders in the Zurich Cohort Study: A twenty year prospective study.. Epidemiol. Psichiatr. Soc..

[38] Vieta E. (2016). DSM-5.1.. Acta Psychiatr. Scand..

[39] Kessing LV, González-Pinto A, Fagiolini A, Bechdolf A, Reif A, Yildiz A, Etain B, Henry C, Severus E, Reininghaus EZ, Morken G, Goodwin GM, Scott J, Geddes JR, Rietschel M, Landén M, Manchia M, Bauer M, Martinez-Cengotitabengoa M, Andreassen OA, Ritter P, Kupka R, Licht RW, Nielsen RE, Schulze TG, Hajek T, Lagerberg TV, Bergink V, Vieta E (2021). DSM-5 and ICD-11 criteria for bipolar disorder: Implications for the prevalence of bipolar disorder and validity of the diagnosis - A narrative review from the ECNP bipolar disorders network.. Eur Neuropsychopharmacol.

[40] Pacchiarotti I., Bond D.J., Baldessarini R.J., Nolen W.A., Grunze H., Licht R.W., Post R.M., Berk M., Goodwin G.M., Sachs G.S., Tondo L., Findling R.L., Youngstrom E.A., Tohen M., Undurraga J., González-Pinto A., Goldberg J.F., Yildiz A., Altshuler L.L., Calabrese J.R., Mitchell P.B., Thase M.E., Koukopoulos A., Colom F., Frye M.A., Malhi G.S., Fountoulakis K.N., Vázquez G., Perlis R.H., Ketter T.A., Cassidy F., Akiskal H., Azorin J.M., Valentí M., Mazzei D.H., Lafer B., Kato T., Mazzarini L., Martínez-Aran A., Parker G., Souery D., Ozerdem A., McElroy S.L., Girardi P., Bauer M., Yatham L.N., Zarate C.A., Nierenberg A.A., Birmaher B., Kanba S., El-Mallakh R.S., Serretti A., Rihmer Z., Young A.H., Kotzalidis G.D., MacQueen G.M., Bowden C.L., Ghaemi S.N., Lopez-Jaramillo C., Rybakowski J., Ha K., Perugi G., Kasper S., Amsterdam J.D., Hirschfeld R.M., Kapczinski F., Vieta E. (2013). The International Society for Bipolar Disorders (ISBD) task force report on antidepressant use in bipolar disorders.. Am. J. Psychiatry.

